# Capturing needles in haystacks: a comparison of B-cell receptor sequencing methods

**DOI:** 10.1186/s12865-014-0029-0

**Published:** 2014-08-05

**Authors:** Rachael JM Bashford-Rogers, Anne L Palser, Saad F Idris, Lisa Carter, Michael Epstein, Robin E Callard, Daniel C Douek, George S Vassiliou, George A Follows, Mike Hubank, Paul Kellam

**Affiliations:** Wellcome Trust Sanger Institute, Wellcome Trust Genome Campus, Hinxton, Cambridge, CB10 1SA UK; Molecular Haematology and Cancer Biology Unit, UCL Institute of Child Health, London, WC1N 1EH UK; Human Immunology Section, Vaccine Research Center, National Institute of Allergy and Infectious Diseases, National Institutes of Health, Bethesda, MD 20892 USA; Department of Hematology, Addenbrooke’s Hospital, Hills Road, Cambridge, CB2 0QQ UK; Research Department of Infection, Division of Infection and Immunity, University College London, Gower Street, London, WC1E 6BT UK

## Abstract

**Background:**

Deep-sequencing methods are rapidly developing in the field of B-cell receptor (BCR) and T-cell receptor (TCR) diversity. These promise to revolutionise our understanding of adaptive immune dynamics, identify novel antibodies, and allow monitoring of minimal residual disease. However, different methods for BCR and TCR enrichment and amplification have been proposed. Here we perform the first systematic comparison between different methods of enrichment, amplification and sequencing for generating BCR and TCR repertoires using large sample numbers.

**Results:**

Resampling from the same RNA or cDNA pool results in highly correlated and reproducible repertoires, but resampling low frequency clones leads to stochastic variance. Repertoires generated by different sequencing methods (454 Roche and Illumina MiSeq) and amplification methods (multiplex PCR, 5’ Rapid amplification of cDNA ends (5’RACE), and RNA-capture) are highly correlated, and resulting IgHV gene frequencies between the different methods were not significantly different. Read length has an impact on captured repertoire structure, and ultimately full-length BCR sequences are most informative for repertoire analysis as diversity outside of the CDR is very useful for phylogenetic analysis. Additionally, we show RNA-based BCR repertoires are more informative than using DNA.

**Conclusions:**

Repertoires generated by different sequencing and amplification methods are consistent, but we show that read lengths, depths and error profiles should be considered in experimental design, and multiple sampling approaches could be employed to minimise stochastic sampling variation. This detailed investigation of immune repertoire sequencing methods is essential for informing basic and clinical research.

**Electronic supplementary material:**

The online version of this article (doi:10.1186/s12865-014-0029-0) contains supplementary material, which is available to authorized users.

## Background

The adaptive immune response selectively expands B- and T-cell clones from a diverse antigen naïve repertoire following antigen recognition by the hyper-variable regions of B- or T-cell receptors (BCR and TCR) respectively [1,2]. Functional BCRs and TCRs are generated by site-specific recombination of V, (D), and J gene segments [3–5], with imprecise joining of the gene segments leading to random deletion and insertion of nucleotides at the junctional regions. Clonal affinity selection for enhanced BCR-antigen or TCR-peptide binding contributes to shaping the mature immune repertoire [6–8].

Mapping of BCR and TCR repertoires promises to transform our understanding of adaptive immune dynamics, with applications ranging from identifying novel antibodies and determining evolutionary pathways for haematological malignancies to monitoring of minimal residual disease following chemotherapy [1,2,8,9]. However, there is concern over the validity of biological insights gained from the different BCR and TCR enrichment, amplification and sequencing methods. With immune repertoire sequencing becoming an increasingly recognised and important tool for understanding the adaptive immune system, we have performed the first systematic comparison between different isolation, amplification and sequencing methods for elucidating B-cell repertoire diversity by deep sequencing. We have used samples of diverse B-cell populations from healthy peripheral blood (PB), clonal B-cell populations from lymphoblastoid cell lines (LCL) and PB from chronic lymphocytic leukaemia (CLL) patients [9]. We have applied a number of approaches to assess the differences between methods. Firstly, IgHV gene usage is typically reported as an assessment of BCR repertoire structure, where healthy individuals exhibit low frequencies of most or all IgHV genes, and where clonal populations have significantly higher frequencies of a single IgHV gene or group of IgHV genes [10]. We formally assess whether there is differential or biased method-specific amplification of each IgHV gene by comparing IgHV frequencies observed between different methods applied to each sample. Secondly, we compare the individual BCR full-length sequence frequencies between different samples to assess the reproducibility of each BCR repertoire method. Thirdly, the overall clonality of each sample can be assessed and compared using previously published clonality measures of vertex Gini indices, cluster Gini indices and maximum cluster sizes using BCR sequence network analysis [9]. Briefly, the Gini index is a measure of unevenness. When applied to the vertex size distribution for a given sample, the Gini index indicates the overall clonal nature of a sample, and when applied to the cluster size distribution, the Gini index indicates the overall somatic hypermutation in the sample. Low vertex Gini indices represent diverse populations and high vertex Gini indices represent clonal populations of B-cells. Similarly, low cluster Gini indices represent populations with lower mutational diversity and high cluster Gini indices represent clonal populations with higher mutational diversity. The maximum cluster size is the percentage of reads corresponding to the largest cluster and indicates the degree of clonal expansion of a sample. This allows assessment of whether overall BCR repertoire structures are faithfully retained between the different methods.

## Methods

### Samples

Peripheral blood mononuclear cells (PBMCs) were isolated from 10 ml of whole blood from 9 healthy volunteers and 8 CLL patients using Ficoll gradients (GE Healthcare). Total RNA was isolated using TRIzol® (Invitrogen) and purified using RNeasy Mini Kit (Qiagen) including on-column DNase digestion according to manufacturer’s instructions. Total RNA was also isolated from 1×10^6^ cells from 10 human lymphoblastoid cell lines (LCLs) from the HapMap project [11]. Research was approved by relevant institutional review boards and ethics committees (07/MRE05/44, Eastern NHS Multi Research Ethics Committee), and all subjects gave written consent for the research [9]. Samples are summarised in Additional file 1: Table S4.

### RNA and DNA multiplex PCR amplification

Multiplex PCR amplification of RNA samples were performed according to Bashford-Rogers et al. [9] (primers in Additional file 1: Table S3). For multiplex PCR amplification of DNA, 30 ng of DNA was mixed with the JH reverse primer and the FR1 forward primer set (0.25 μM each), using 0.5 μl Phusion® High-Fidelity DNA Polymerase (Finnzymes), 1 μl dNTPs (0.25 mM), 1 μl DTT (0.25 mM), per 50 μl reaction. The following PCR program was used: 3 minutes at 94°C, 35 cycles of 30 seconds at 94°C, 30 seconds at 60°C and 1 minute at 72°C, with a final extension cycle of 7 minutes at 72°C on an MJ Thermocycler.

### RNA-capture

Total RNA was initially processed for target enrichment using the NEBNext kit (NEB) according to manufacturers protocol. Briefly, mRNA was isolated by polyA + selection and converted to cDNA. cDNA at 0.3 to 0.7 ng/μl was fragmented to 200 bp (Covaris), ligated to sequencing adaptors (Illumina) and size selected at 200 bp (Life Technologies E-Gel). Samples were then indexed for pre-capture pooling (NEBNext Multiplex Oligos for Illumina Index Primers 1 to 12). A pre-capture library was generated using 12 cycles of PCR (KAPA Biosystems Library Amplification Kit). Libraries were pooled and hybridised to biotinylated RNA-capture baits (custom design [12], full protocol available on request), Agilent SureSelect) at 65°C for 24 h. Hybridised fragments were selected using streptavidin magnetic beads, washed and eluted for multiplexed sequencing on Illumina Miseq.

### 5’RACE

5’RACE was performed using SMARTer™ Pico PCR cDNA Synthesis Kit (Clontech) according to Clontech protocols, using the JH-reverse primer (Additional file 1: Table S3) and SMARTer 5’ primer for PCR amplification.

### Sequencing methods and read preparation

Sequencing libraries were prepared using standard Roche-454 Rapid Prep or Illumina protocols, and sequenced using an FLX Titanium Genome Sequencer (Roche/454 Life Sciences) or by 250 bp paired-ended MiSeq (Illumina) respectively. Raw 454 or MiSeq reads were filtered for base quality (median Phred score >32) using the QUASR program. (http://sourceforge.net/projects/quasr/) [13]. The 250 bp reads from the 5’RACE experiment were retained if they contained a JH-reverse primer sequence and orientated to begin with IgHV gene. Non-immunoglobulin sequences were removed and only reads with significant similarity to reference IgHV genes from the IMGT database [14] using BLAST [15] were retained (1×10^−10^ E-value threshold). Primer sequences were trimmed from the reads, and sequences retained for analysis only if both primer sequences were identified. Reads from RNA-capture were BLAST aligned to reference IgH genes, and trimmed if the reads extended outside the IgHV-D-J region. Reads from each platform were filtered for length (>255 bp, 120 bp and 160 bp for 454, MiSeq (250 bp paired-end) and RNA-capture MiSeq respectively). The combined per-base error-rate for the RT-PCR and sequencing process for the 454 and MiSeq platforms were similar to other studies (1.74x10^−4^ and 2.06×10^−4^ respectively) [9,10,16]. Excluding homopolymeric indels, the per-base error rate for 454 is 7.04×10^−5^.

### Repertoire analysis

For identification of IgHV genes, BLAST [15] was used to align the BCR sequences against known BCR sequences from the ImMunoGeneTics (IMGT) database [14] (e-value threshold of 10^−20^). Network assemblies and diversity measure calculations (vertex Gini index, cluster Gini index and maximum cluster size) were performed according to Bashford-Rogers *et al.* [9]. Statistical analyses were performed in R. Differences between 454 sequence sets excluded homopolymeric indels.

### Simulation of sampling BCR populations

For a given sequencing depth *N*, the range of values, *x,* within 10% of the true BCR proportion *p*_*i*_ would be

$$ {b}_{lower}\le x\ \le {b}_{upper} $$

Where *b*_*lower*_ = *N* * *p*_*i*_ * 0.9 *b*_*lower*_ = *N* * *p*_*i*_ * 0.9 and *b*_*upper*_ = *N* * *p*_*i*_ * 1.1, and 0 ≤ *b*_*lower*_, *b*_*upper*_ ≤ *N*. With a sequencing error rate *e* per base, the probability of successfully sequencing the BCR sequence of length *l* becomes *p* = *p*_*i*_ − (*e* * *l*). Therefore the probability of sampling within the range *x* is the sum of the binomial probabilities of the range *x*:

$$ P(x) = {\displaystyle \sum_{i={b}_{lower}}^{b_{upper}}}\left(\begin{array}{c}\hfill N\hfill \\ {}\hfill i\hfill \end{array}\right)\ {p}^i{\left(1-p\right)}^{N-i} $$

To estimate the probability of sequencing at least one read of a given type, the Poisson distribution can be employed:

$$ P\left(X\ne 0\right) = 1-{e}^{-\lambda } $$

Where *λ* is the expected value of sequencing reads of that type, *λ* = *N* * *p*.

## Results and discussion

### Assessing the stochasticity of sampling B-cell repertoires

**Figure 1:**
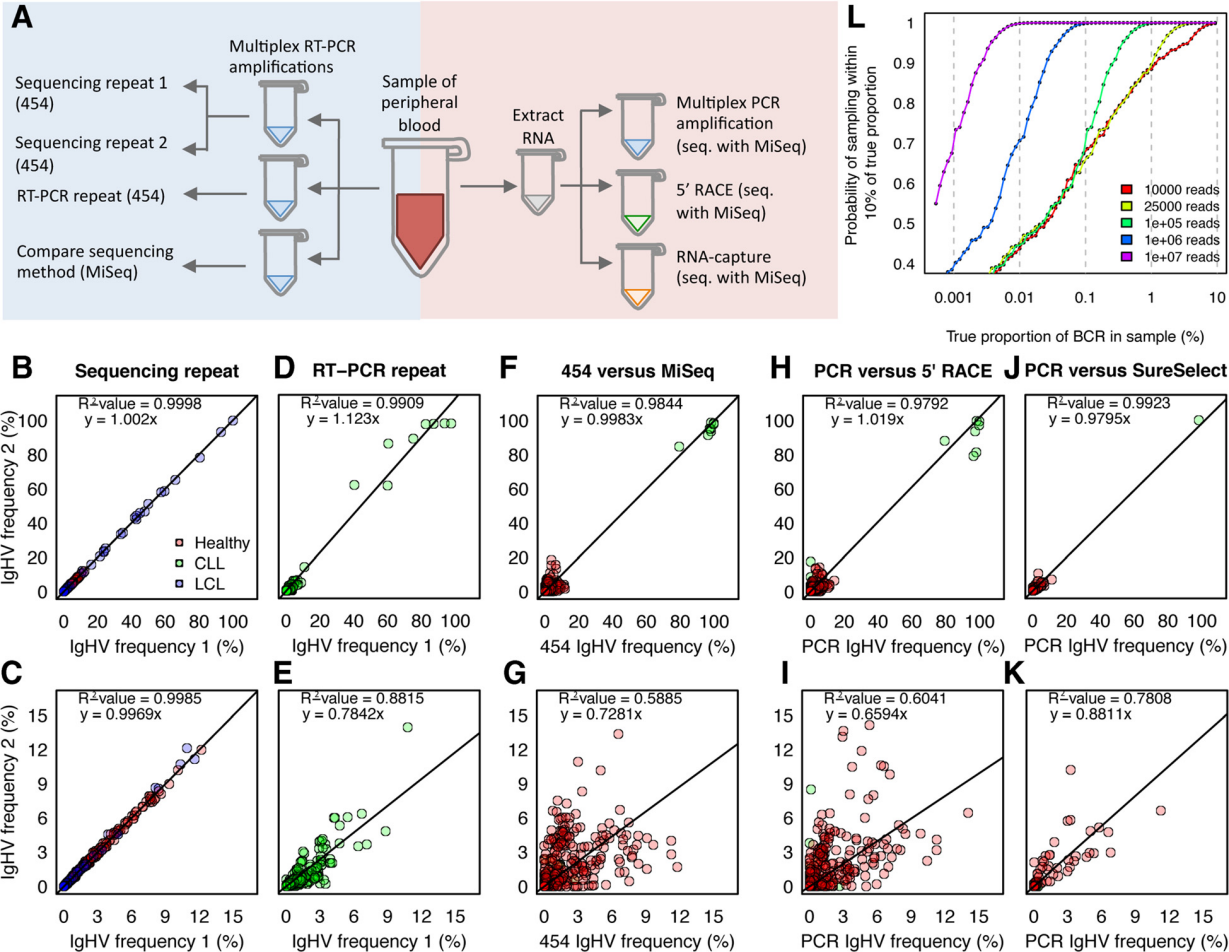
**Comparing different RNA-capture and amplification methods. A)** Schematic diagram of all experiments. Left side: RNA was extracted from B-cell samples, and multiplex RT-PCR performed in triplicate: sequencing repeats (re-sequencing the same PCR products), PCR repeats (independent RT-PCR of the same RNA and sequencing by MiSeq) and sequencing method comparisons (independent RT-PCR of the same RNA source and sequenced by 454 and MiSeq). Right side: RNA was extracted from B-cell samples, and 5’RACE (by MiSeq), RNA-capture (by MiSeq) were compared to PCR amplification of the same samples (using 454 sequencing). Graphs of IgHV gene-usage frequency distributions between samples were generated from **B)** the sequencing repeats, **D)** RT-PCR repeats, **F)** sequencing method comparisons, **H)** multiplex PCR versus 5’RACE (by MiSeq), **J)** multiplex PCR versus RNA-capture (sequenced by MiSeq). Graphs **C, E, G, I** and **K)** are IgHV gene-usage frequency distributions from only the low frequencies (<15%) respectively. Point colors are red, blue and green for healthy, LCL and CLL samples respectively. The linear regression equation and R^2^-values are given. **L)** Plot of the probability of sampling within 10% of the true of a BCR proportion with varying read depths (10,000, 25,000, 100,000, 1,000,000 and 10,000,000 reads) assuming an initial population of 50,000,000 BCR sequences after amplification.

As exhaustive sampling of B-cells is not possible in humans, the “true” extent of the BCR repertoire in humans can only be estimated. A typical PB sample (10-20 ml) accounts for ~0.2% of the total PB, from which only a fraction is used in current BCR sequencing methods. Therefore, we examined the effect of resampling on repertoire structure. Firstly, we repeated repertoire sequencing of the same multiplex PCR products from 10 LCL and 5 healthy PB samples using 454 sequencing (Figure 1A, sequencing repeat). Comparing frequency distributions for each IgHV gene formally assesses differential representation of particular IgHV genes. The IgHV frequencies are highly correlated between repeats with a gradient close to unitary (Figure 1B, R^2^-value = 0.9998, y = 1.002×, unitary gradient equals a one-to-one mapping between repeats) even at low IgHV frequencies (Figure 1C), suggesting minimal stochasticity introduced through sequencing alone.

Next, we determined the stochastic variation observed when re-sampling 2 equimolar portions of the same RNA from 9 CLL PB samples, and repeating both PCR and MiSeq sequencing (300 bp paired-end, Figure 1A, RT-PCR repeats). The IgHV frequencies were again highly correlated (R^2^-value = 0.9909, y = 1.115x, Figure 1D). The correlation is lower than the sequencing repeats suggesting greater re-sampling stochasticity introduced at the PCR steps. As the correlation might be skewed by the very high clonality of the CLL samples, the expected correlation between experimental conditions using diverse samples is best assessed from low frequency gene usage, shown in Figure 1E. As expected, the correlation between IgHV genes present at low frequencies (<15%, representing frequencies typically observed in diverse B-cell samples) is less than that of IgHVs present at higher frequency reflecting lower probabilities of re-sampling rarer molecules (R^2^-value = 0.8815 for RT-PCR repeats, Figure 1E), which is in-line with previous studies [9].

We also used clonality measures, Gini index and maximum cluster size, and individual BCR sequence frequencies to determine whether overall BCR repertoire structures were faithfully retained between the repeats [9]. These diversity measures correspond to that seen in equivalent sample types in previous studies (Table 1, [9]). These repertoire diversity measures are strongly correlated between both sequencing and RT-PCR repeats (Additional file 1: Figure S2 A-B, R^2^-values > 0.991). We show that the correlation between the individual BCR frequencies between RT-PCR repeats is strong (R^2^ = 0.9793), although again the correlation is weaker when considering only the low frequency BCRs (Additional file 1: Figure S2 C). Therefore, samples from the same RNA pool exhibit some re-sampling stochasticity, particularly for low frequency variants. However, overall repeated samples are highly correlated and repertoires are reproducible.

**Table 1:** **Mean diversity measures for each sample type**

**Sample type**	**Mean maximum cluster size (%)**	**Mean vertex Gini index**	**Mean cluster Gini index**
**Healthy**	0.581	0.182	0.047
**Chronic lymphocytic leukemia (CLL)**	95.117	0.931	0.612
**Human lymphoblastoid cell line (LCL)**	65.205	0.934	0.790

### Assessing differences between sequencing methods

Different sequencing platforms each have different read-lengths, depths and error profiles (Additional file 1: Tables S1-2). 454 sequencing uses emulsion PCR and pyrosequencing and can produce reads potentially over 800 bp [17], and therefore has the capacity to sequence a full BCR amplicon in a single read. However, the 454 platform has high homopolymeric base pair error-rates caused by accumulated light intensity variance [16,18–20]. The Illumina MiSeq has the highest throughput per run (1.6 Gb of sequence/run, 60 Mb/hour) [17] and lower overall error rate, particularly in homopolymeric regions [21]. MiSeq however has its own distinct error profile of single-base errors associated with GGC motif [22] and at the 3’ end of the reads compared to the 5’ end. MiSeq can currently generate up to 300 bp paired-end reads that allows for paired-end joining and full coverage of multiplex PCR amplicons. We compared sequencing technologies by taking two portions of RNA from 8 CLL and 6 healthy PB samples and performed PCR followed by 454 or MiSeq (250 bp paired-end) sequencing (Figure 1A, sequencing comparison). The IgHV frequencies between the sequencing methods were highly correlated (R^2^-value = 0.9844, y = 0.998x, Figure 1F). As the correlation might be skewed by the very high clonality of the CLL samples, we assessed the correlation at low frequency gene usages. Again, greater variation of low frequency variants suggests both effects of stochastic re-sampling and platform-specific differences (gene frequencies <15% representing typical observations from diverse B-cell samples, Figure 1G, R^2^-value = 0.5885). The individual BCR sequence frequencies were also highly correlated (Additional file 1: Figure S3A), suggesting that repertoire structure is retained when using the same amplification method on different sequencing platforms. However, due to the lower homopolymeric indel rate, only the MiSeq platform is currently appropriate for filtering read sets for open reading frames (and subsequent translation into protein sequence). MiSeq also has the advantage of a higher sequencing depth per lane, therefore allowing higher levels of multiplexing of samples and reducing the per-sample cost.

### How deep do we need to sequence?

The sequencing depth required depends on the frequencies of clones of interest and sequencing method. Reads from all methods were filtered for quality and presence of immunoglobulin sequence as detailed in the methods section. Here, the percentage of filtered BCR sequences from PCR amplification was 60% and 76% for MiSeq and 454 sequencing respectively and 55% for 5’RACE (using MiSeq). As the RNA-capture baits target both BCRs and TCRs, the percentage of usable BCR sequences was only 1.53% (Additional file 1: Table S2). Therefore, between 35-50x higher sequencing depth is required for RNA-capture to obtain the equivalent number of BCR-specific reads compared to the other methods. To determine the number of BCR sequences required for biological studies, we modelled the probabilities of sequencing BCR clones at varying BCR sampling proportions and sequencing depths. Assuming an initial population of 50,000,000 BCRs after amplification, when a BCR clone is >4% of the total population, a sequencing depth of only 10,000 reads has a 95% probability of sequencing within 90% accuracy (i.e. within 10% of the true clonal proportion, Figure 1L). For rarer BCR clones, higher sequencing depths can significantly increase sampling accuracy. For example, the probability of sequencing within 90% accuracy for a clone at 0.04% of the total population is increased from 0.522 at 100,000 reads to 0.956 at 1,000,000 reads (i.e. 1/10 lane of MiSeq). For clones of <0.001%, increasing the sequencing depth to as high as 1×10^7^ does not significantly increase sequencing accuracy due to low re-sampling probabilities. Thus, the optimum sequencing depth depends on the samples used and biological question. Studies investigating highly clonal disorders, such as CLL, require less reads to obtain information about clonal sequence than studies of healthy individuals with diverse repertoires of low frequency clones.

### Assessing different RNA-capture and amplification methods

The three main BCR amplification methods are PCR using IgH-specific multiplex primers [23], 5’ Rapid amplification of cDNA ends (5’RACE) [24–27] and RNA-capture using RNA bait/capture probes [28,29]. IgH-specific multiplex PCR primers have been designed [23], validated [30–34], and used in numerous biological studies [9,10,35–41]. Such multiplex PCRs can be performed on either RNA or DNA and require a relatively small amount of template. However, there is the potential for biased primer annealing and unequal PCR amplification of BCR sequences. RNA-capture is based around the methods used for human exome sequencing and uses RNA bait/capture probes and subsequent universal PCR amplification [28,29]. This allows for enrichment, amplification and sequencing of TCRs (α, β, γ and δ chains) and BCRs (heavy and light chains) simultaneously. PCR and RNA-capture methods can use RNA or DNA, but have the potential for sequence-based differential annealing and biased capture. 5’RACE overcomes this by using a single IgH-specific primer for first strand Ig cDNA synthesis and subsequent sequence-independent template switching primer for second strand cDNA synthesis. This eliminates potential multiplex primer bias, but can have low efficiency, high non-specific amplification, and short fragment contamination from RNA degradation or incomplete cDNA synthesis and template switching [24–27]. Also, as the RNA bait probes and multiplex PCR primers are generated from reference Ig and TCR gene databases, they lack the same efficiency as 5’RACE for capturing human allelic variants of TCR or BCR segments that are not represented in the reference database.

To compare the different amplification methods, 5’RACE (with MiSeq sequencing) was performed on 7 CLL and 5 healthy PB samples, RNA-capture (with MiSeq sequencing) was performed on 1 healthy and 1 CLL PB, and were compared to multiplex PCR of the same samples (using 454 sequencing, Figure 1A). Strong IgHV gene frequency correlations were observed between PCR and 5’RACE (R^2^-value = 0.9792), and between PCR and RNA-capture (R^2^-value = 0.9795) (Figures 1H-K). This correlation is again weaker for lower frequency BCR sequences (R^2^-value = 0.6041 and 0.8811 respectively, Figures 1I and K). Comparing individual BCR sequences rather than IgHV gene frequencies showed strong correlations between all the methods (R^2^-value > 0.96, Additional file 1: Figure S3) above 5% BCR sequence frequency. Both Pairwise-Wilcoxon tests and paired T-tests between IgHV gene frequencies (with Bonferroni multiple-testing corrections) showed no significant differentially captured IgHV genes between the RNA-capture, 5’RACE or PCR methods. Together, we suggest each method here captures similar BCR repertoires.

### Effect of amplicon length

**Figure 2:**
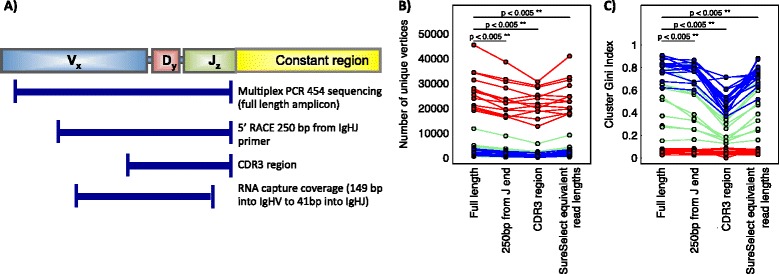
**Variation of diversity measures with read-length. A)** Schematic diagram showing the read-lengths from each technique aligned against the BCR gene. 454 multiplex sequencing reads were trimmed either between i) containing bases within 250 bp from the end of the IgHJ region, ii) CDR3 region, iii) or the mean region covered by reads from the RNA-capture method (149 bp from 3’end of IgHV to 41 bp from 5’end of IgHJ), and corresponding BCR networks were generated. Plots show the variation of **B)** number of unique sequencing reads and **C)** Cluster Gini Index. Point colors are red, green and blue for healthy PBMC, LCL and CLL samples respectively.

Shorter amplicons give less phylogenetic information and mutational pathways of B-cell clones may be lost, thus artificially separating related BCRs into different clusters. Within B-cell networks different BCR sequences can be reduced into the same vertex if the mutations are located outside the read, so clusters have lower numbers of vertices. Therefore, we compared the impact of using different length amplicons on the diversity of the generated BCR repertoire. The PCR sequencing reads were trimmed to represent three regions of the IgH molecule: i) sequences containing bases within 250 bp from the end of the IgHJ region (mimicking reads from the 5’RACE experiment), ii) sequences covering the most variable part of the IgH molecule, the complementarity-determining region 3 (CDR3), that is often the focus of biological studies, such as [42,43], or iii) the mean region covered by reads from RNA-capture (~170 bp, between ~115 bp from the IgHV 3’ end and ~30 bp from the IgHJ 5’ end)), (Figure 2A and Additional file 1: Figure S4). The corresponding BCR sequence networks were generated. The number of unique BCR sequences per sample reduced significantly from 10847 using the full-length PCR reads to 9555, 8041, 8974 using 5’RACE-equivalent, CDR3 and RNA-capture read-lengths respectively (p-values < 0.005, Figure 2B). The diversity of the resulting networks using cluster Gini indices show significant deviation from the full-length PCR reads (Figure 2C, visualized in Additional file 1: Figure S5). Using sequencing platforms with shorter read lengths, e.g. Illumina with less than 250 bp reads also lower the potential to capture IgH genetic diversity, thus reducing repertoire information. The diversity outside of CDR3 is very useful to capture for phylogenetic analysis and ultimately the full-length BCR sequence (obtainable from 300 bp paired-ended MiSeq reads or by 454 sequencing) is most informative for repertoire analysis.

### RNA versus DNA: which is best for BCR sequencing?

**Figure 3:**
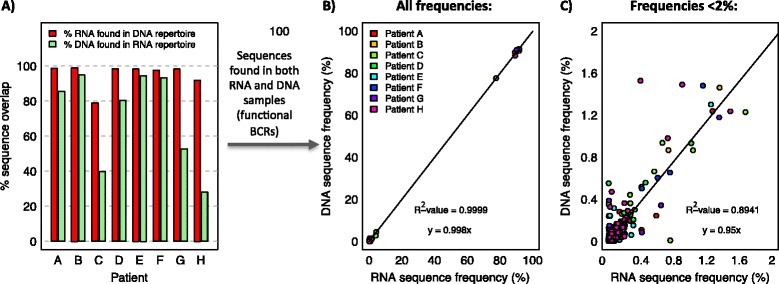
**Comparison of RNA and DNA repertoires.** RNA and DNA were extracted from each peripheral blood sample from 8 CLL patients, on which multiplex RT-PCR or PCR was performed respectively and sequenced by MiSeq (250 bp paired-end). **A)** The percentage of DNA sequences found in each RNA sample. The correlation between the BCR frequency in RNA and functional DNA repertoires (DNA sequences that were found also in the RNA repertoire) for the 8 CLL patients in **B)** all IgHV gene usage frequencies and **C)** the low frequency IgHV gene usage frequencies only (<2%). If unequal numbers of RNA molecules per cell significantly skewed the RNA BCR repertoires, then deviation from y = x correlation would be expected.

PCR and MiSeq (250 bp, paired-end) sequencing was performed on both RNA and DNA fractions from 8 CLL patients’ PB to compare the effect of input material. First BCR allele defective-rearrangements present in the genomic DNA have the potential to artificially increase the number of clones in the data [44], whereas unequal numbers of RNA molecules per cell may skew the BCR repertoires derived from RNA. An average of 71.2% of reads from the DNA repertoire were represented at least once in the RNA repertoire (range 28.1-94.9%, Figure 3A). Sequences found in both RNA and DNA repertoires are likely to be functional BCR sequences, whereas DNA sequences not observed in the RNA repertoire could either be non-functional by the process of “allelic-exclusion”, or due to the lack of re-sampling. The frequencies of individual BCR sequences from RNA compared to the functional DNA reads (i.e. DNA reads found in the RNA repertoire) are strongly correlated (R^2^-value = 0.9999, y = 0.988x) suggesting no repertoire-skewing between the DNA and RNA proportions, even at low frequencies (Figure 3B-C). Therefore, due to defective-rearrangements present in the genomic DNA, RNA is potentially more informative than DNA for understanding BCR population structures.

## Conclusions

Next-generation sequencing of immune receptor genes can provide a quantitative understanding of the landscape of the adaptive immune response. The “true” BCR repertoire in humans is not known, and current methods rely on taking small samples of the total B-cells to estimate the population structure. Here we show little sampling bias in repeat samples and that multiplex PCR, RNA-capture and 5’RACE each captures a similar overall BCR repertoire and clonal features of each sample. RNA capture offers the advantage of capturing both B and T-cell repertoires. We show that there is no significant inflation or deflation of clonality due to unequal numbers of RNA transcripts per cell and suggest that using RNA input is more informative for understanding B-cell population structure as genomic DNA potentially exhibits artificially increased numbers of clones reflecting biallelic rearrangements in a single clone [44]. Choice of sequencing platform does not significantly affect the repertoire structure captured but an amplicon and sequence reads covering the entire BCR is most informative for analysis and sequencing depth should be sufficient to allow capture of the BCR frequency of interest. The ability to detect BCR repertoire diversity and sensitivity varies with read length and depth respectively, resulting in an ideal BCR sequencing solution of amplification of the full VDJ region to a depth of 1,000,000 to identify unique BCRs at 0.04% frequency with 90% theoretical accuracy.

We show that the repertoires generated by different sequencing and amplification methods is robust but read lengths, depths and error profiles should be considered in experimental design and multiple sampling approaches could be employed to minimise stochastic sampling issues. We consider the multiplex PCR method to be the most automatable and sensitive method, with consistently good amplification from samples with low numbers of B-cells. The number of PCR cycles can be tailored to the requirement of DNA amount required for sequencing, and therefore the best method for large studies or using samples with low cell numbers. We recommend the use of 5’RACE if a sample is likely to be highly somatically mutated, thus potentially modifying the annealing sites for the multiplex PCR or RNA capture. However, we have shown that in CLL, where there is ongoing somatic hypermutation, we see no evidence of differential primer annealing ability. RNA-capture can be useful for situations where both the B- and T-cell repertoires are to be assessed simultaneously. For sequencing, we recommend MiSeq as it is able to produce high quality reads covering the full BCR, with read depths allowing for sequencing of many samples on a single run by multiplexing.

## Additional file

Additional file 1:
**Supplementary information.** Description of data: Supplemental data file.

## Abbreviations

BCR: B-cell receptor
TCR: T-cell receptor
PB: Peripheral blood
CLL: Chronic lymphocytic leukemia
5’RACE: 5’ Rapid amplification of cDNA ends
LCL: Lymphoblastoid cell line
