# Supplementary material for: Capturing needles in haystacks: a comparison of B-cell receptor sequencing methods
Source: BMC Immunol. 2014 Aug 5;15:29. doi: 10.1186/s12865-014-0029-0 (PMC4243823; doi:10.1186/s12865-014-0029-0)
Supplement: Additional file 1: — Supplementary information. Description of data: Supplemental data file. [file 12865_2014_29_MOESM1_ESM.pdf]

Supplementary information

Table S1. Technical information of the next-generation sequencing platforms used in this study.

Sequencing platform	454 sequencing	Illumina MiSeq sequencing
**Read Length**	Up to 800 bp	2x150 or 2x 250bp paired end
**Typical Throughput ***	35 Mb	1.5-4.5 Gb
**Reads per Run**	~$1x10^6$ reads	~$1x10^7$ reads
**Run Time**	23 hours	16-24 hours
**Indels per 100 bp ****	0.4011	0.0009
**Substitutions per 100 bp ****	0.0543	0.0921

* From: Quail, MA. et al. 2012. A tale of three next generation sequencing platforms: comparison of Ion Torrent, Pacific Biosciences and Illumina MiSeq sequencers[1].

**From: Junemann, S. et al. 2013. Updating benchtop sequencing performance comparison[2].

Table S2. Mean and standard deviation of read depths per sample and the average number of samples multiplexed per lane/run for each of the 454 and MiSeq sample sets.

Technology	Mean read depth per sample (after filtering)	Average number of multiplexed samples per lane/run	Average % BCR sequences after filtering*
Multiplex PCR (454)	33,413	12	76.10
Multiplex PCR (MiSeq)	31,118	50	60.30
5' RACE (MiSeq)	72,586	95	55.09
RNA capture (MiSeq)	58,015	2	1.53

* Percentage of reads after filtering for functionally rearranged BCR sequences from the whole read set. RNA capture has lower percentage of filtered reads due to the designed simultaneous capture of immunoglobulin heavy and light chains as well as T-cell receptors.

Table S3. Primers used in each method.

Primer	Sequence	
JH reverse	CTTACCTGAGGAGACGGTGACC	
VH1-FR1 forward	GGCCTCAGTGAAGGTCTCCTGCAAG	
VH2-FR1 forward	GTCTGGTCCTACGCTGGTGAAACCC	
VH3-FR1 forward	CTGGGGGGTCCCTGAGACTCTCCTG	FR1 primer set*
VH4-FR1 forward	CTTCGGAGACCCTGTCCCTCACCTG	
VH5-FR1 forward	CGGGGAGTCTCTGAACATCTCCTGT	
VH6-FR1 forward	TCGCAGACCCTCTCACTCACCTGTG	
VH1-FR2 forward	CTGGGTGCGACAGGCCCCTGGACAA	
VH2-FR2 forward	TGGATCCGTCAGCCCCCAGGGAAGG	
VH3-FR2 forward	GGTCCGCCAGGCTCCAGGGAA	
VH4-FR2 forward	TGGATCCGCCAGCCCCCAGGGAAGG	FR2 primer set*
VH5-FR2 forward	GGGTGCGCCAGATGCCCGGGAAAGG	
VH6-FR2 forward	TGGATCAGGCAGTCCCCATCGAGAG	
VH7-FR2 forward	TTGGGTGCGACAGGCCCCTGGACAA	
B-actin forward	CGCCTTTGCCGATCCGCCG	
B-actin reverse	CTTCTCGCGGTTGGCCTTGGG	
GAPDH forward	GAAGGTGAAGGTCGGAGTC	
GAPDH reverse	GAAGATGGTGATGGGATTTC	
B-globin forward	CTGCCGTTACTGCCCTGTGGG	
B-globin reverse	GGACAGCAAGAAAGCGAGCTTAGTG	

* From: van Dongen, J. J. et al. Design and standardization of PCR primers and protocols for detection of clonal immunoglobulin and T-cell receptor gene recombinations in suspect lymphoproliferations: report of the BIOMED-2 Concerted Action BMH4-CT98-3936. [3]

Table S4. Samples used in this study for each amplification method.

Sample type*	ID	Multiplex (454)	Multiplex (MiSeq)	5' RACE (MiSeq)	RNA capture (MiSeq)
CLL	Sample 1	Y	Y	Y	Y
CLL	Sample 2	Y	Y	Y	-
CLL	Sample 3	Y	Y	-	-
CLL	Sample 4	Y	Y	Y	-
CLL	Sample 5	Y	Y	Y	-
CLL	Sample 6	Y	Y	Y	-
CLL	Sample 7	Y	Y	Y	-
CLL	Sample 8	Y	Y	Y	-
Healthy	Sample A	Y	-	Y	-
Healthy	Sample B	Y	-	Y	-
Healthy	Sample C	Y	Y	Y	-
Healthy	Sample D	Y	-	Y	-
Healthy	Sample E	Y	Y	Y	-
Healthy	Sample F	Y	Y	-	-
Healthy	Sample G	Y	Y	-	-
Healthy	Sample H	Y	Y	-	-
Healthy	Sample I	Y	Y	-	Y
LCL	LCL 1	Y	-	-	-
LCL	LCL 2	Y	-	-	-
LCL	LCL 3	Y	-	-	-
LCL	LCL 4	Y	-	-	-
LCL	LCL 5	Y	-	-	-
LCL	LCL 6	Y	-	-	-
LCL	LCL 7	Y	-	-	-
LCL	LCL 8	Y	-	-	-
LCL	LCL 9	Y	-	-	-
LCL	LCL 10	Y	-	-	-

*RT-PCR and sequencing repeats were performed using multiplex PCR and MiSeq sequencing on human lymphoblastoid cell line (LCL) and healthy samples

** Abbreviations: CLL = chronic lymphocytic leukemia, LCL = human lymphoblastoid cell line.

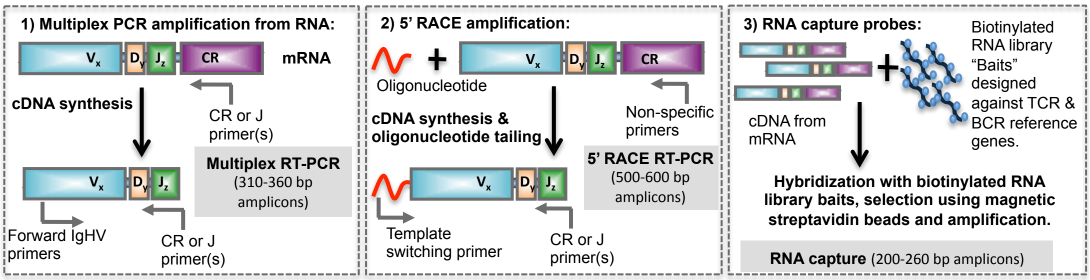

Figure S1. Different IgH RNA sequencing methods. A schematic diagram of the different methods: RNA was extracted from peripheral blood samples and multiplex PCR (sequenced by both 454 and MiSeq), 5'RACE (sequenced by 250bp paired-end MiSeq) and RNA-capture (sequenced by MiSeq) was performed. Multiplex RT-PCR of RNA uses degenerate primers located in conserved regions of the IgHV and IgHJ genes. 5' Rapid amplification of cDNA ends (5'RACE) of RNA uses a single IgHJ or constant region primer and a template switching primer. RNA-capture uses RNA bait probes and subsequent PCR amplification.

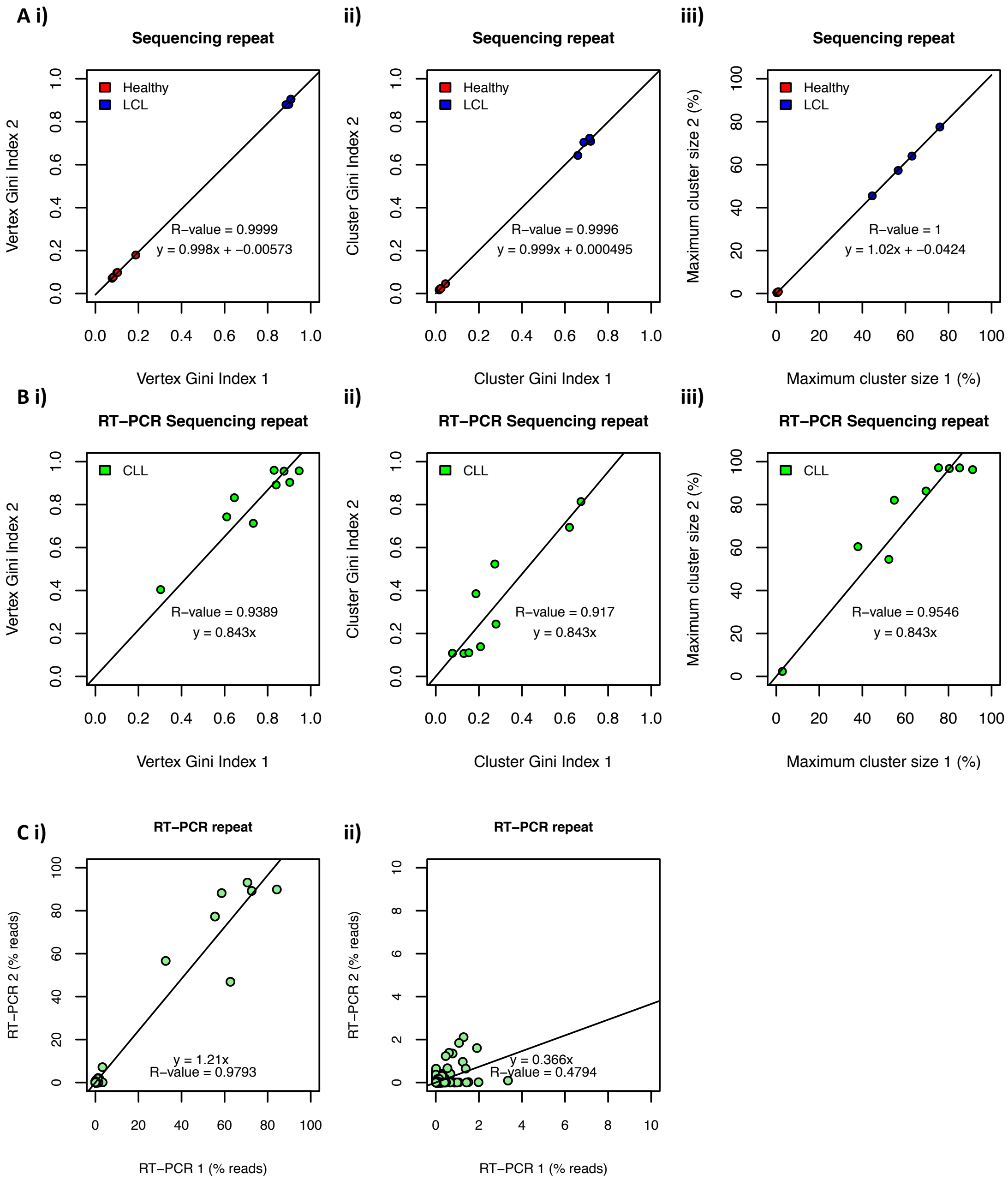

Figure S2. Diversity measure correlations between technical repeats. Sequencing repeats (sequencing the same PCR products twice) were performed on 5 healthy peripheral blood (PB) and 10 human lymphoblastoid cell line (LCL) samples. PCR repeats (independent RT-PCR of the same RNA and sequencing by 454) were performed on 9 CLL samples. BCR sequence networks were generated from the BCR sequences and graphs of diversity measures between samples were generated from **A)** the sequencing repeats and **B)** the RT-PCR repeats for **i)** Vertex Gini index, **ii)** Cluster Gini index and **iii)** maximum cluster size. Point colors are red, green and blue for healthy PBMC, LCL and CLL samples respectively. **C)** Individual BCR frequencies between RT-PCR repeats, where **C i)** shows all the BCRs, and **C ii)** shows only the low frequency BCRs (<10%).

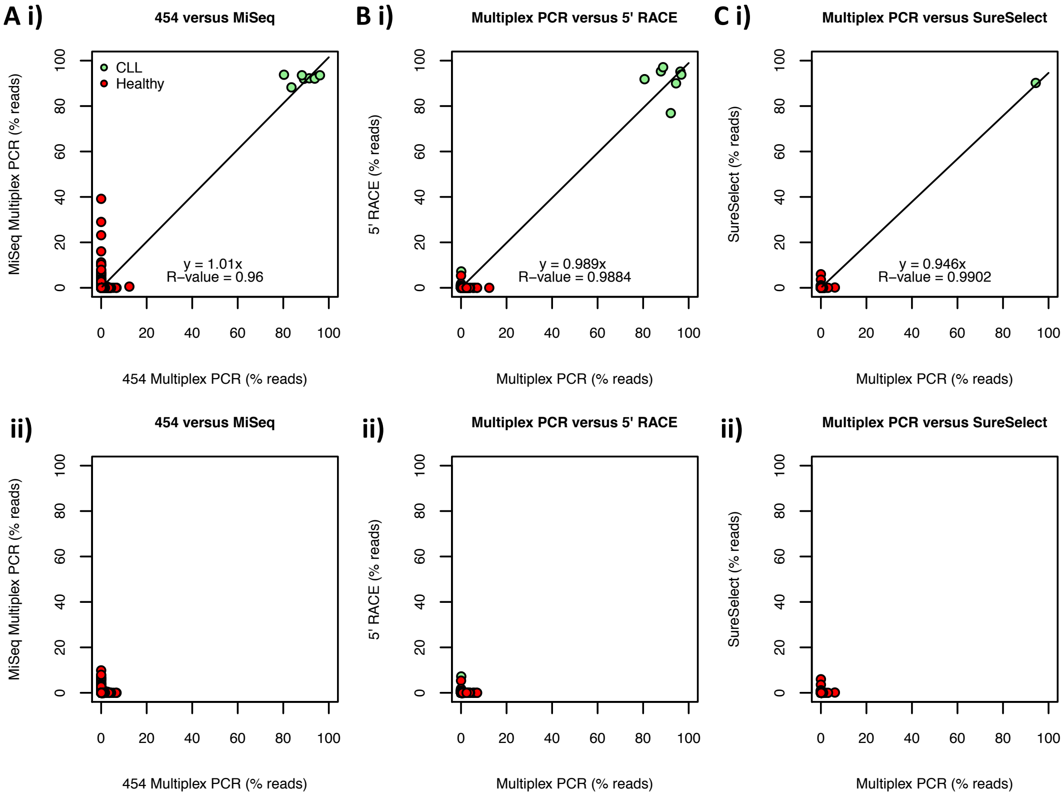

Figure S3. Comparison of BCR frequencies of all individual BCR sequences for each sample between three methods: A) 454 versus MiSeq multiplex, **B)** Multiplex PCR (by 454) against 5' RACE (by MiSeq) and **C)** Multiplex PCR (by 454) against RNA-capture (by MiSeq). The plots labeled (i) show the whole data, and the plots labeled (ii) show the low frequency BCRs only (<10%). Point colors are red and green for healthy PBMC and CLL samples respectively.

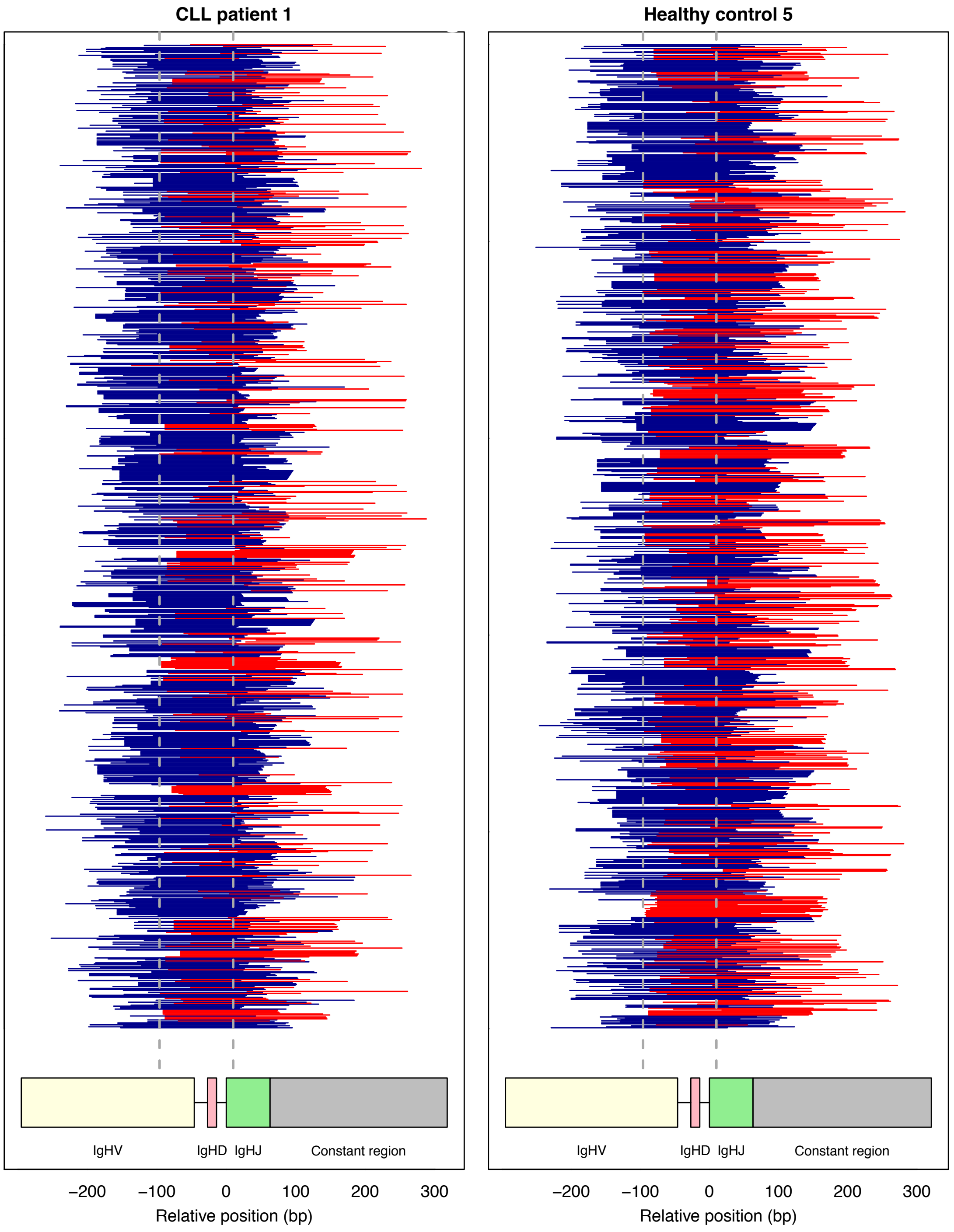

Figure S4. Alignment of RNA capture reads to BCR sequence. The reads that allow for adequate IgHV, D, J classifications are shown in blue (68.9% of all IgH specific reads), and the remainder shown in red.

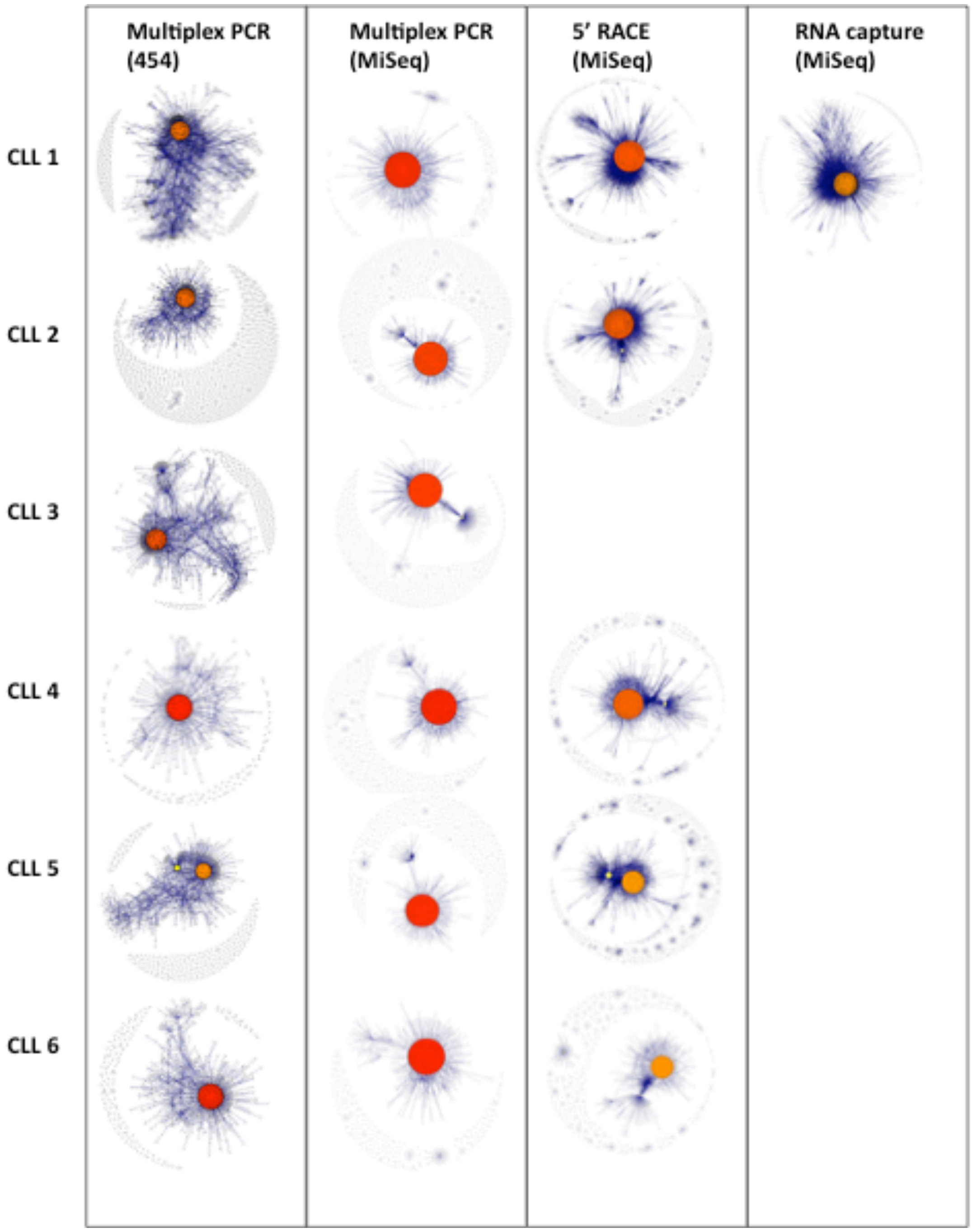
Multiplex PCR (454)
Multiplex PCR (MiSeq)
5' RACE (MiSeq)
RNA capture (MiSeq)
CLL 1
CLL 2
CLL 3
CLL 4
CLL 5
CLL 6

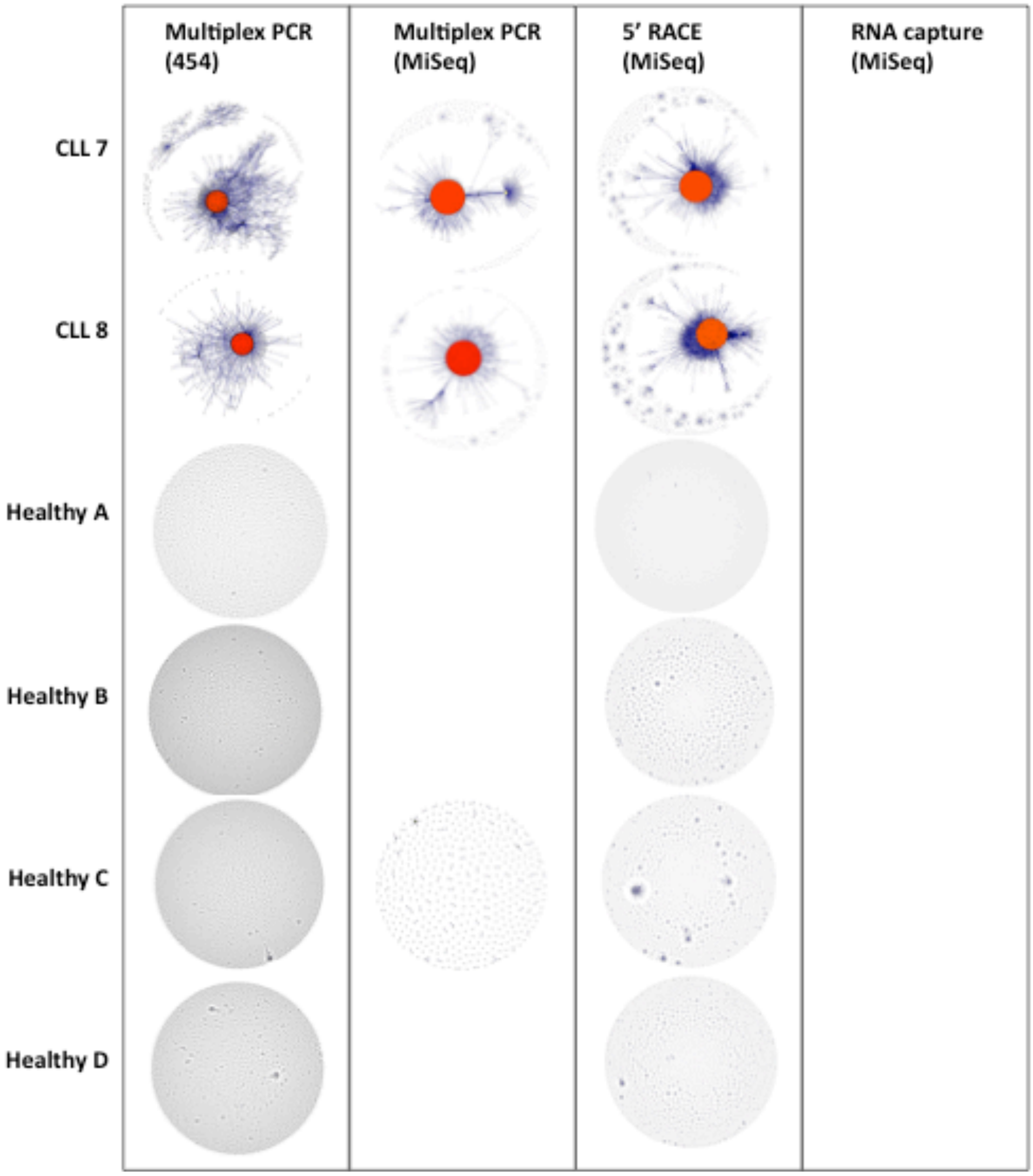
Multiplex PCR (454)
Multiplex PCR (MiSeq)
5' RACE (MiSeq)
RNA capture (MiSeq)
CLL 7
CLL 8
Healthy A
Healthy B
Healthy C
Healthy D

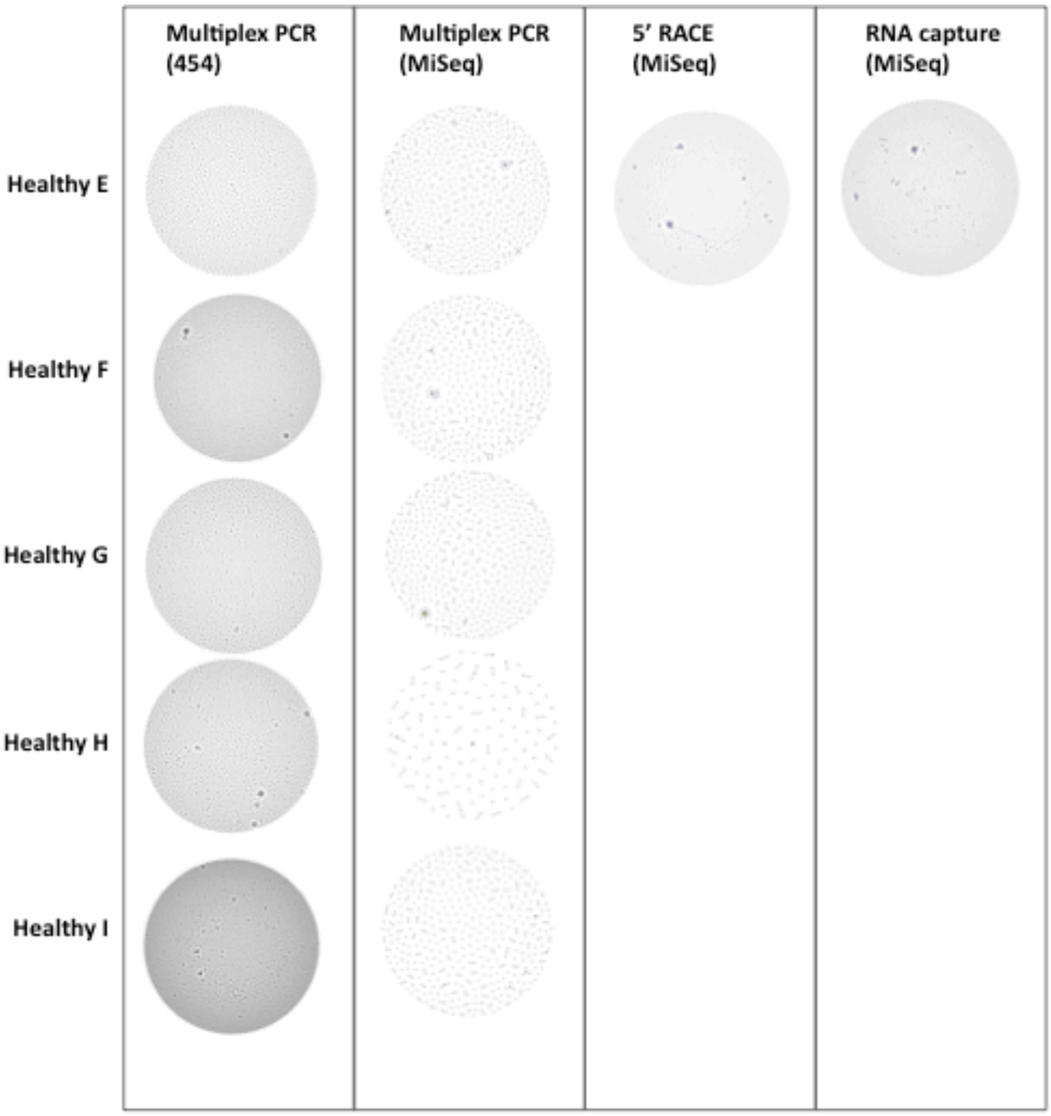

Figure S5. Visual representation of the BCR sequence networks for all the CLL and healthy samples in this study ordered by technique. BCR sequence network generation was performed according to Bashford-Rogers *et al.*[4]. Each vertex represents a unique sequence, and the number of identical BCR sequences defines the vertex size. Edges are created between vertices that differ by one nucleotide (either non-homopolymeric indels or substitutions). The groups of interconnected vertices (clusters) comprise of similarly re-arranged BCRs.

References:

1 Quail, M. A. *et al.* A tale of three next generation sequencing platforms: comparison of Ion Torrent, Pacific Biosciences and Illumina MiSeq sequencers. *BMC genomics* **13**, 341, doi:10.1186/1471-2164-13-341 (2012).

2 Junemann, S. *et al.* Updating benchtop sequencing performance comparison. *Nat Biotechnol* **31**, 294-296, doi:10.1038/nbt.2522 (2013).

3 van Dongen, J. J. *et al.* Design and standardization of PCR primers and protocols for detection of clonal immunoglobulin and T-cell receptor gene recombinations in suspect lymphoproliferations: report of the BIOMED-2 Concerted Action BMH4-CT98-3936. *Leukemia* **17**, 2257-2317, doi:10.1038/sj.leu.2403202 (2003).

4 Bashford-Rogers, R. J. *et al.* Network properties derived from deep sequencing of human B-cell receptor repertoires delineate B-cell populations. *Genome Res*, doi:10.1101/gr.154815.113 (2013).
